# Glia, sympathetic activity and cardiovascular disease

**DOI:** 10.1113/EP085713

**Published:** 2016-04-09

**Authors:** Nephtali Marina, Anja G. Teschemacher, Sergey Kasparov, Alexander V. Gourine

**Affiliations:** ^1^ Department of Clinical PharmacologyUniversity College LondonLondonWC1E 6JFUK; ^2^ School of Physiology and Pharmacology, Medical Sciences Building, Bristol Heart InstituteUniversity of BristolBristolBS8 1TDUK; ^3^ Centre for Cardiovascular and Metabolic Neuroscience, Neuroscience, Physiology & PharmacologyUniversity College LondonLondonWC1E 6BTUK

## Abstract

**New Findings:**

- **What is the topic of this review?**
  In this review, we discuss recent findings that provide a novel insight into the mechanisms that link glial cell function with the pathogenesis of cardiovascular disease, including systemic arterial hypertension and chronic heart failure.
- **What advances does it highlight?**
  We discuss how glial cells may influence central presympathetic circuits, leading to maladaptive and detrimental increases in sympathetic activity and contributing to the development and progression of cardiovascular disease.

Increased activity of the sympathetic nervous system is associated with the development of cardiovascular disease and may contribute to its progression. Vasomotor and cardiac sympathetic activities are generated by the neuronal circuits located in the hypothalamus and the brainstem. These neuronal networks receive multiple inputs from the periphery and other parts of the CNS and, at a local level, may be influenced by their non‐neuronal neighbours, in particular glial cells. In this review, we discuss recent experimental evidence suggesting that astrocytes and microglial cells are able to modulate the activity of sympathoexcitatory neural networks in disparate physiological and pathophysiological conditions. We focus on the chemosensory properties of astrocytes residing in the rostral ventrolateral medulla oblongata and discuss signalling mechanisms leading to glial activation during brain hypoxia and inflammation. Alterations in these mechanisms may lead to heightened activity of sympathoexcitatory CNS circuits and contribute to maladaptive and detrimental increases in sympathetic tone associated with systemic arterial hypertension and chronic heart failure.

## Introduction

Cardiovascular disease remains the most common cause of death in Western societies, although the relative mortality attributable to it has decreased significantly in the last decade (Go *et al*. 2013). Hypertension is an important risk factor for the development of cardiovascular disease, and recent statistics from the American Heart Association suggest that approximately one‐third of adults in the USA are hypertensive (Go *et al*. 2013). Despite significant progress in the prevention, diagnosis and treatment of hypertension, only half of patients show satisfactory response to treatment (Go *et al*. 2013). Inadequate adherence to antihypertensive medication is an important factor that affects disease management. However, relatively poor efficacy of the existing antihypertensive therapies might also be due to the fact that conventional treatments target the peripheral mechanisms that maintain high systemic blood pressure, whereas primary CNS factors are responsible for the development and progression of the disease and remain untreated.

The pathophysiology of cardiovascular disease is complex, multifactorial and, in many respects, poorly understood. In this review, we focus on essential hypertension and chronic heart failure, which are conditions known to be associated with significant changes in the central nervous mechanisms of cardiovascular control. Indeed, recent advances in autonomic nervous system monitoring techniques in humans (including microneurography, measurement of noradrenaline spillover, assessment of heart rate variability and baroreflex sensitivity) have shown that increased activity of the sympathetic nervous system is intimately linked with the development and progression of both essential hypertension and chronic heart failure (Hasking *et al*. 1986; Leimbach *et al*. 1986; Esler *et al*. 1989; Kaye *et al*. 1995; Grassi *et al*. 1998; La Rovere *et al*. 1998, 2003; Esler & Kaye, 2000; Esler *et al*. 2001). In these conditions, complex interactions between behavioural, nutritional and humoral factors, altered activity of cardiovascular afferents (baro‐ and chemoreceptors) and modifications in the central nervous control mechanisms all lead to increased sympathetic efferent activity (Vallbo *et al*. 1979; Trzebski *et al*. 1982; Grassi *et al*. 1988, 2002; Mark, 1990; van de Borne *et al*. 1997; Schultz & Sun, 2000; Carlyle *et al*. 2002; Schultz *et al*. 2007; Grassi, 2009; Siński *et al*. 2012; May *et al*. 2013). In hypertension, enhanced sympathetic tone, along with humoral and vascular factors, is believed to play a significant role in the development and maintenance of high arterial blood pressure. In heart failure, sympathetic drive increases in parallel with the progression of the disease as a compensatory measure aimed to preserve ventricular contractile function. However, in the long‐term, sustained elevation of sympathetic tone becomes maladaptive and detrimental, leading to morphological and functional changes in the peripheral vasculature and the myocardium, including hypertrophy and proliferation of smooth muscle cells (Bevan, 1984), increased arterial stiffness (Boutouyrie *et al*. 1994), endothelial dysfunction (Pettersson *et al*. 1990), atherosclerosis (Kaplan *et al*. 1987), increased left ventricular mass (Simpson, 1983) and increased arrhythmogenesis (Lown & Verrier, 1976). Understanding the CNS mechanisms that underlie the increases in activity of the sympathetic nervous system is, therefore, important for the development of novel therapeutic strategies to treat hypertension and heart failure, and may ultimately help to reduce the clinical, social and economic burden of cardiovascular disease.

Vasomotor and cardiac neural activities of spinal sympathetic preganglionic neurones depend on tonic descending excitatory drive generated by sympathoexcitatory (presympathetic) neuronal circuits residing in the brainstem and the hypothalamus (Dampney, 1994; Spyer, 1994; Guyenet, 2000; Dampney *et al*. 2003; Madden & Sved, 2003). These circuits include the rostral ventrolateral medulla (RVLM), rostral ventromedial and mid‐line medulla, the A5 cell group of the pons and the hypothalamic paraventricular nucleus (PVN; Strack *et al*. 1989; Dampney *et al*. 2003; Madden & Sved, 2003; Kanbar *et al*. 2011; Marina *et al*. 2011). Although RVLM neurones responsible for sympathetic control of cardiovascular activities have been studied extensively (see Guyenet, 2013), little or no attention has been paid to their non‐neuronal neighbours, astrocytes and microglia. Here, we review recent evidence suggesting that changes in astroglial and microglial activities contribute to modifications in central nervous autonomic control and, therefore, may play an important role in the development and progression of cardiovascular disease associated with increased activity of the sympathetic nervous system (Marina, 2013; Marina, 2015; Rana *et al*. 2010; Shi *et al*. 2010; Zubcevic *et al*. 2011).

## Glial cells

Astrocytes are the most abundant CNS glial cells and occupy non‐overlapping territories that define their functional domains (Halassa *et al*. 2007). Astrocytes were traditionally considered as a rather passive CNS cellular component that assisted neuronal circuits to maintain their function by providing nutritional and structural support. However, significant evidence accumulated in the last two decades suggests that astrocytes may play an active role in the regulation of synaptic strength and information processing (Halassa *et al*. 2009). In pathological conditions, astrocytes respond to CNS injury with significant changes in gene expression, morphology and function. This process, called astrogliosis, might have both beneficial and detrimental effects on tissue repair and might potentially influence the activity of neuronal networks (Sofroniew, 2015).

Microglial cells are resident immune cells in the CNS involved in the detection of invading organisms and brain injury. Microglial cells are less abundant than astrocytes, making up ∼20% of all brain glial cells. In resting conditions, microglial cells present a ramified morphology characterized by a small soma and extensive branched and long processes, which might make direct contact with neurones, astrocytes and cerebral vasculature. In response to brain injury, microglial cells become activated, undergo significant morphological changes and release various substances that may exert either neuroprotective or neurotoxic effects (Badoer, 2010).

## Gliotransmission

Significant experimental evidence indicates that communication between glial cells and CNS neurones is bidirectional in nature. Astrocytes are capable of controlling synaptic communication via complex interactions with presynaptic and postsynaptic elements (Perea *et al*. 2009). Astrocytes release (glio)transmitters in response to activation of G‐protein‐coupled receptors and, when activated, generate intracellular Ca^2+^ responses. Several molecules identified as gliotransmitters can be released by activated astrocytes, including ATP/adenosine, d‐serine, glutamate, GABA, l‐lactate and possibly some others (Volterra & Meldolesi, 2005; Holmström *et al*. 2013; Tang *et al*. 2014; Marina *et al*. 2015).

One well‐documented astroglial signalling mechanism involves the release of ATP. The ATP released by activated astrocytes into the extracellular space acts on purinergic (P2) metabotropic and ionotropic receptors expressed by adjacent astrocytes, neurones and other glial cells (Gourine *et al*. 2009). Ectonucleotidases break down ATP to ADP, AMP and adenosine, which in turn can activate a different group of G‐protein‐coupled adenosine (P1) receptors (Burnstock, 2007).

Although release of (glio)transmitter(s) can be triggered by many stimuli, the physiological significance of gliotransmission has been debated. According to some estimates, the amount of glutamate contained within astroglial ‘vesicles’ is not sufficiently high to trigger changes in the neuronal activity following its release (Bramham *et al*. 1990). Indeed, the functional significance of gliotransmission is difficult to demonstrate because the approaches commonly used to stimulate astrocytes and to interfere with gliotransmitter release are generally non‐specific (Hamilton & Attwell, 2010). When genetic methods of selective activation and blockade of astrocytic Gq G‐protein‐coupled receptor‐mediated Ca^2+^ signalling mechanisms were applied in animal models, neuronal excitatory synaptic transmission in the hippocampus, as well as short‐ and long‐term plasticity were found to be unaffected (Fiacco *et al*. 2007; Agulhon *et al*. 2010). This may be explained by the fact that mature astrocytes do not express metabotropic glutamate receptor 5, thus casting further doubt on the role of glutamate in mediating communication between neurones and astrocytes (Sun *et al*. 2013). These results emphasize the importance of ATP‐mediated signalling by astrocytes, which is further supported by the recent studies demonstrating that ATP release by brainstem astrocytes plays an important role in the CNS mechanisms that maintain cardiovascular and respiratory homeostasis.

## Glial cells and central chemosensitivity

There is significant evidence that astrocytes are involved in central chemosensory mechanisms that maintain cardiorespiratory homeostasis (Gourine *et al*. 2010; Marina *et al*. 2013). In mammals, these mechanisms are vital in producing adaptive changes in breathing in response to changes in $P_{CO_{2}}$/pH and $P_{O_{2}}$ in the arterial blood and brain parenchyma. Systemic hypercapnia, leading to decreases in blood and brain pH, is associated with a rapid release of ATP within the chemosensory areas of the brainstem (Gourine *et al*. 2005 *b*). Interestingly, these chemosensitive areas are located immediately underneath and in close proximity to the populations of RVLM presympathetic and ventral respiratory column neurones, which generate co‐ordinated sympathetic and respiratory rhythms (Gourine *et al*. 2005 *a*,*b*; 2010). Hypercapnia‐induced ATP release is independent of the peripheral chemoreceptor inputs (Gourine *et al*. 2005 *b*) and occurs within the appropriate time frame and in sufficient quantities to be responsible for the subsequent increases in breathing. Consistent with this, blockade of ATP receptors at the sites of release reduces the CO_2_‐induced increase in breathing, whereas application of ATP evokes an increase in the respiratory activity, mimicking the effect of CO_2_ (Gourine *et al*. 2005 *b*). Recent studies have also provided evidence that brainstem astrocytes are exquisitely sensitive to changes in pH within the physiological range and may represent the main source of ATP released in the brainstem during hypercapnia (Gourine *et al*. 2010). In contrast to cortical astrocytes, ventral brainstem astrocytes respond to acidification with elevations in intracellular Ca^2+^ and an increased rate of exocytosis of ATP‐containing vesicular compartments (Kasymov *et al*. 2013). This suggests that ventral brainstem astrocytes are functionally specialized for monitoring physiological increases in $P_{CO_{2}}$/[H^+^] and responding to these increases with the release of ATP (Kasymov *et al*. 2013). ATP propagates Ca^2+^ excitation among neighbouring astrocytes, activates neurones of the ventral respiratory column and, thus, contributes to the adaptive increases in the respiratory activity (Gourine *et al*. 2010).

**Figure 1:**
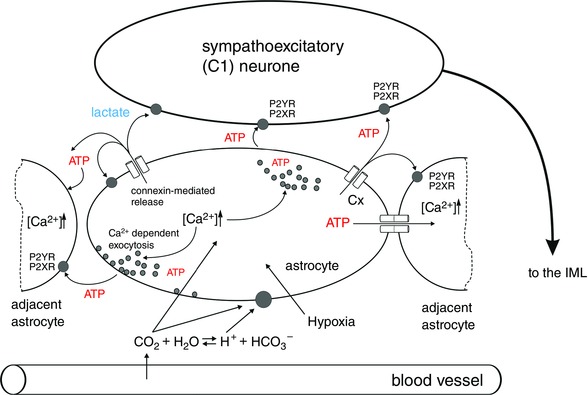
**Neuroglial interactions in the rostral ventrolateral medulla oblongata hypothesized to underlie pathological increases in sympathetic nerve activity in chronic heart failure and essential hypertension** Tissue hypoxia and increased concentration of CO_2_/H^+^ is detected by astrocytes, leading to the release of ATP and lactate, which in turn increase the excitability of bulbospinal sympathoexcitatory (C1) neurones. Abbreviations: Cx, connexin; and IML, intermediolateral cell column.

Astrocytes also appear to be sensitive to physiological changes in brain O_2_ content. They respond to decreases in $P_{O_{2}}$ a few millimetres of mercury below normal brain oxygenation with robust increases in intracellular [Ca^2+^]. Hypoxia is detected in mitochondria, where O_2_ is consumed (Angelova *et al*. 2015). Decreases in $P_{O_{2}}$ inhibit mitochondrial respiration, leading to mitochondrial depolarization, production of free radicals, lipid peroxidation, activation of phospholipase C and Inositol triphosphate (IP_3_) receptors, resulting in recruitment of Ca^2+^ from the intracellular stores and vesicular release of ATP (Angelova *et al*. 2015; Fig. 1). This hypoxia‐induced astroglial ATP release has been demonstrated to maintain enhanced respiratory activity even in the absence of oxygen sensing by the peripheral chemoreceptors (Angelova *et al*. 2015).

## Role of brainstem glia in the pathogenesis of cardiovascular disease

New evidence suggests that glial dysfunction may be responsible for the pathogenesis of certain neurodevelopmental disorders previously attributed to primary neuronal abnormalities. For example, recent studies demonstrated that altered astroglial and microglial functions contribute to the pathogenesis of Rett syndrome (Lioy *et al*. 2011; Derecki *et al*. 2012; Okabe *et al*. 2012), a prototypical neurological disorder associated with mutations of the methyl‐CpG‐binding protein 2 (*MECP2*) gene, which is manifested by neurological, respiratory and autonomic deficiencies. In this section, we discuss putative mechanisms that link altered glial cell activity with maladaptive increases in excitability of CNS presympathetic circuits, which may contribute to the detrimental sympathetic activation in cardiovascular disease.

### Hypoxia

The (patho)physiological conditions associated with activation of RVLM astrocytes and downstream responses of presympathetic circuits are still unclear, although this mechanism may become significant in conditions of central hypoxia, which is a powerful stimulus for the release of ATP and lactate in the RVLM (Gourine *et al*. 2005 *b*; Karagiannis *et al*. 2015). ATP was previously shown to be released from within the RVLM in response to hypoxia (10% inspired O_2_, 5 min). This release was only slightly attenuated in animals with bilateral sectioning of vagi, aortic and carotid sinus nerves, suggesting that most of the hypoxia‐induced ATP release originates from astrocytes. It was also found that the amount of hypoxia‐induced ATP released from the ventral medullary structures is similar to that released in response to CO_2_ (Gourine *et al*. 2005 *a*). Activation of ATP receptors in the RVLM by microinjections of ATP or stable ATP analogues has been shown to activate bulbospinal presympathetic neurones, leading to marked increases in the arterial blood pressure, heart rate and renal sympathetic nerve activity (Sun *et al*. 1992; Horiuchi *et al*. 1999; Ralevic, 2000). These data are consistent with the hypothesis that increases in the level of ‘ambient’ ATP within the RVLM as a result of hypercapnia and/or hypoxia‐induced activation of brainstem astrocytes may contribute to the increases in central sympathetic drive (Fig. 1).

Factors that trigger and maintain astrocytic activation during the development and progression of heart failure remain unknown. Both obstructive sleep apnoea and central sleep apnoea are common clinical features in heart failure patients (Bradley & Floras, 2003 *a*,*b*). These conditions are associated with recurrent episodes of systemic hypoxia and hyper/hypocapnia. Brain tissue oxygen content in many heart failure patients was found to be significantly decreased despite nearly normal oxygen levels measured in the arterial blood (Rifai *et al*. 2012). Therefore, a hypoxic environment is likely to result in higher extracellular concentrations of ATP within the brainstem and this, as discussed above, may increase the activity of presympathetic neurones and result in higher sympathetic tone (Fig. 1). Indeed, sympathetic tone appears to be significantly higher in heart failure patients with sleep apnoea in comparison to heart failure patients with normal breathing patterns (Naughton *et al*. 1995; Mansfield *et al*. 2003).

Similar mechanisms may also underlie sympathoexcitation associated with the development of systemic arterial hypertension (Marina *et al*. 2015). Earlier studies demonstrated that basilar artery diameter in animal models of hypertension (spontaneously hypertensive rats, SHRs) is reduced, resulting in increased vascular resistance (Cates *et al*. 2011, 2012). Structural changes of brain vasculature are unlikely to be a consequence of hypertension because they are evident in prehypertensive animals (Cates *et al*. 2011, 2012). It was reported recently that tissue $P_{O_{2}}$ in the RVLM of the SHR is lower than in normotensive counterparts (despite normal levels of arterial $P_{O_{2}}$), suggesting that in the SHR the sympathoexcitatory networks are exposed to a hypoxic environment (Marina *et al*. 2015). Thus, in neurogenic hypertension, the Cushing mechanism is hypothesized to maintain brain perfusion by increasing systemic arterial blood pressure. Increased sympathetic activity and systemic hypertension can be viewed as a compensatory mechanism activated to preserve oxygen delivery and maintain brain oxygenation at the expense of systemic hypertension (Rodbard & Stone, 1955; Cates *et al*. 2011, 2012).

**Figure 2:**
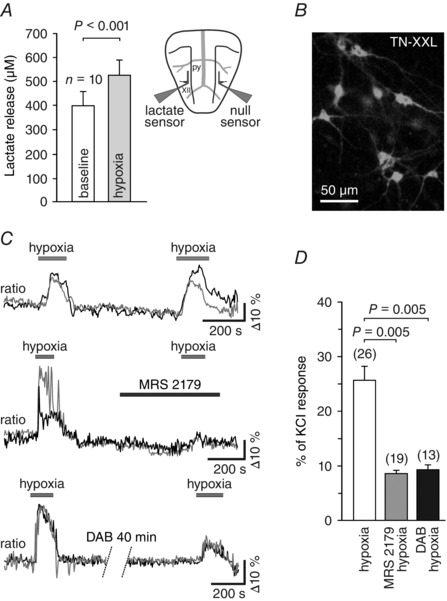
**Release and actions of ATP and lactate mediate activation of rostral ventrolateral medulla (RVLM) neurons during hypoxia** *A*, summary data obtained *in vitro* using horizontal slices of the rat brainstem, showing tonic release of lactate from the ventral surface of the medulla oblongata and peak lactate release during hypoxia. Inset, schematic drawing of a horizontal brainstem slice, illustrating dual recording configuration of lactate and null (control) biosensors placed on the ventral medullary surface. The difference in current between lactate and null biosensors was used to determine the amount of lactate release. Abbreviations: py, pyramidal tract; and XII hypoglossal rootlets. *B*, putative presympathetic C1 RVLM neurons visualized in organotypic brainstem slices after transduction with an adenoviral vector to express genetically encoded Ca^2+^ indicator TN‐XXL under the control of PRSx8 promoter. *C*, raw traces (changes in intracellular [Ca^2+^] of two individual neurons are shown on each plot), illustrating robust and reproducible responses of the RVLM neurons to hypoxia (top traces) as well as the effects of the ATP receptor antagonist MRS2179 (30 μmol l^−1^; middle traces) and the glycogenolysis inhibitor 1,4‐dideoxy‐1,4‐imino‐d‐arabinitol (DAB; 500 μmol l^−1^; bottom traces) on hypoxia‐induced [Ca^2+^]_i_ responses of these neurons (ratiometric imaging using TN‐XXL). *D*, summary data illustrating the effects of MRS2179 and DAB on hypoxia‐induced [Ca^2+^]_i_ responses of putative C1 neurons. Data are presented as means ± SEM. Reproduced from Marina *et al*. (2015).

Brain hypoxia is known to trigger robust activation of the RVLM presympathetic neurones (Sun & Reis, 1994). There is evidence suggesting that the sensitivity of the RVLM presympathetic neurones to decreases in $P_{O_{2}}$ is mostly indirect and mediated by prior release and actions of both ATP and lactate, released by astrocytes (Fig. 2; Marina *et al*. 2015).

In a similar manner to ATP, lactate, which is also released by activated astrocytes (Tang *et al*. 2014), produces strong excitation of C1 neurons *in vitro* and increases in sympathetic nerve activity and the arterial blood pressure when applied on the brainstem surface (Marina *et al*. 2015). Together, these lines of evidence suggest that both heart failure and hypertension are associated with a decrease in brain parenchymal oxygen content, accumulation of ATP and lactate within the presympathetic brainstem areas, leading to increased activity of sympathoexcitatory neurons and concomitant sustained elevation of sympathetic drive (Fig. 1). It remains to be determined whether in the human brain, tissue extracellular ATP and lactate concentrations are correlated with the severity of the sympathetically mediated cardiovascular diseases.

### Increased purinergic (glio)transmission in the brainstem

**Figure 3:**
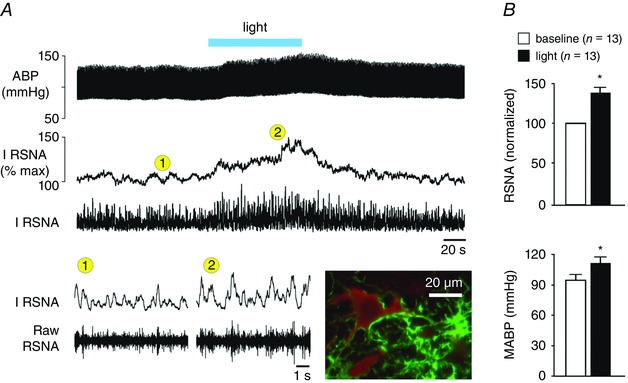
**Optogenetic stimulation of RVLM astrocytes evokes sympathoexcitation *in vivo*** *A*, unilateral optogenetic stimulation of RVLM astrocytes expressing channelrhodopsin 2 increases sympathetic nerve activity and arterial blood pressure in an anaesthetized and artificially ventilated rat. Abbreviations: ABP, arterial blood pressure; I RSNA, integrated renal sympathetic nerve activity; and RSNA, renal sympathetic nerve activity. Inset microphotograph depicts an example of a tyrosine hydroxylase (red immunofluorescence)‐expressing C1 neurone embraced by astrocytic processes expressing channelrhodopsin 2‐Venus (green fluorescence). *B*, summary data illustrating the effect of optogenetic stimulation of RVLM astrocytes on mean arterial blood pressure (MABP) and RSNA. Group data are shown as means + SEM. *P < 0.05 (paired *t*‐test). Reproduced from Marina *et al*. (2013) with permission from Springer.

Activated brainstem astrocytes signal to bulbospinal sympathoexcitatory neurones. It was shown that optogenetic stimulation of RVLM astrocytes (transduced with viral vectors to express light‐sensitive channels, e.g. channelrhodopsin 2) excites presympathetic RVLM neurones belonging to the C1 catecholaminergic group (Marina *et al*. 2013). These responses were attenuated in the presence of an ATP‐degrading enzyme, apyrase, demonstrating a key role for ATP in mediating communication between activated astrocytes and presympathetic neurones (Marina *et al*. 2013). Furthermore, *in vivo* experiments conducted in anaesthetized and artificially ventilated rats showed that optogenetic activation of channelrhodopsin 2‐expressing astrocytes within the RVLM produces robust elevations in renal sympathetic nerve activity, heart rate and arterial blood pressure (Marina *et al*. 2013; Fig. 3). Thus, selective recruitment of [Ca^2+^]_i_ in RVLM astrocytes using an optogenetic approach is sufficient to trigger activation of sympathoexcitatory CNS circuits.

It was also shown in a rat model of heart failure that ATP‐mediated purinergic signalling in the RVLM plays an important role in sympathoexcitation, which is associated with, and may contribute to, the progression of left ventricular remodelling after a myocardial infarction. Given that specific ‘inhibition’ of astroglial activity/function is difficult to achieve (and it is not always easy to choose which of their functions has to be targeted), we reasoned that the role of astrocytes in the control of sympathetic activity in pathological conditions could be investigated by using experimental tools that prevent communication between these cells. To interfere with ATP‐mediated signalling, a lentiviral vector was developed in order to drive expression of a potent ectonucleotidase, transmembrane prostatic acid phosphatase (TMPAP), the effect of which is to facilitate rapid breakdown of extracellular as well as vesicular ATP (Wells *et al*. 2015). The TMPAP was tagged with green fluorescent protein, anchored to the plasma membrane and had a catalytic domain facing the extracellular space. Bilateral overexpression of TMPAP within the RVLM presympathetic circuits reduced sympathetic activity in developing heart failure (evident from a lower plasma concentration of noradrenaline) and slowed the progression of left ventricular remodelling and dysfunction (Marina *et al*. 2013). These data provided the first experimental evidence suggesting that altered glial activity leading to a higher level of ‘ambient’ ATP in the brainstem might be responsible for the increases in sympathetic tone and, by doing so, contribute to progression of heart failure (Fig 1).

Recent studies have demonstrated that ATP‐mediated purinergic signalling in the RVLM may also play a role in the development of neurogenic hypertension. Facilitated breakdown of ATP with targeted overexpression of virally driven TMPAP in the RVLM resulted in a significant reduction of the systemic arterial blood pressure in the SHR (Marina *et al*. 2015). There is also recent evidence to suggest that ATP‐mediated signalling may contribute to alterations in the central nervous mechanisms of autonomic control and development of hypertension in a rat model of chronic intermittent hypoxia. Increases in sympathetic nerve discharge evoked by microinjections of ATP into the RVLM were found to be significantly higher in rats exposed to chronic intermittent hypoxia (Zoccal *et al*. 2011). Chronic intermittent hypoxia was also associated with a significant upregulation of ATP receptor (P2X_3_ and P2X_4_ subunits in particular) expression in the RVLM (Zoccal *et al*. 2011). It remains to be determined whether increased ATP‐mediated signalling in the RVLM in a rat model of chronic intermittent hypoxia originates from activation of astrocytes in response to recurrent changes in the brainstem tissue $P_{O_{2}}$ (Angelova *et al*. 2015).

### Microglial cell activation, astrogliosis and inflammation

Subtle chronic inflammation is a hallmark feature in the pathogenesis of chronic heart failure and essential hypertension. Indeed, in an animal model of heart failure, the expression of the pro‐inflammatory cytokines interleukin‐1β (IL‐1β) and tumour necrosis factor‐α (TNF‐α) was found to be significantly increased in at least one brain site that controls sympathetic outflow, namely the hypothalamic paraventricular nucleus (Kang *et al*. 2008, 2009). Clear evidence of microglial activation within the PVN after myocardial infarction was also reported (Rana *et al*. 2010).

Experimental models of hypertension (rats receiving chronic infusion of angiotensin II) provided further evidence of microglial activation and enhanced production of pro‐inflammatory cytokines in the presympathetic regions of the brain (PVN in particular; Shi *et al*. 2010). Activated microglial cells and inflammatory cytokines can evoke and amplify gliotransmitter release by astrocytes (Cotrina *et al*. 2000; Bezzi *et al*. 2001; Domercq *et al*. 2006; Pascual *et al*. 2012). Interestingly, in the model of hypertension induced by angiotensin II infusion, intracerebroventricular application of an anti‐inflammatory antibiotic, minocyclin, decreased the number of activated microglial cells and reduced the expression of pro‐inflammatory cytokines in the PVN. Suppression of microglial activity and PVN inflammation was associated with lower systemic arterial blood pressure, attenuated ventricular hypertrophy and lower plasma noradrenaline, all of which are indicative of a reduced sympathetic activity (Shi *et al*. 2010).

Immunohistochemical studies have also revealed microglial activation in the PVN of SHRs (Shi *et al*. 2010; Zubcevic *et al*. 2011). Recent data demonstrated that cultured hypothalamic microglial cells are directly activated by prorenin, a component of the renin–angiotensin system, resulting in induction of pro‐inflammatory mechanisms in these cells via activation of nuclear factor‐κB (Shi *et al*. 2014). It remains to be determined whether heart failure and hypertension are associated with similar changes in microglial function in other CNS areas involved in cardiovascular control.

There is also significant evidence to suggest that in pathological conditions, astrogliosis may lead to altered gliotransmission, which may affect neuronal excitability and result in neurotoxicity. Chronic astroglial activation occurs in response to an increase in extracellular ATP as well as in response to the actions of inflammatory cytokines, including IL‐1β and TNF‐α (Bezzi *et al*. 2001; Liu *et al*. 2011; Pascual *et al*. 2012), released locally by activated microglia. For example, in acute hippocampal slices, activated microglia release small amounts of ATP, which triggers waves of Ca^2+^ excitation in astrocytic networks (via stimulation of P2Y_1_ receptors), leading to the release of glutamate and an increased frequency of excitatory postsynaptic events in adjacent neurones (Pascual *et al*. 2012). Furthermore, TNF‐α released by activated microglia induces further release of TNF‐α by local astrocytes. These interactions between activated micro‐ and astroglia result in a significant production of TNF‐α, followed by a release of glutamate (Bezzi *et al*. 2001). All these factors are capable of initiating propagating waves of Ca^2+^ excitation within astroglial networks and modulating the excitability of local neuronal circuits (Cotrina *et al*. 2000; Bezzi *et al*. 2001; Pascual *et al*. 2012). Thus, in pathological conditions, the activity of CNS neuronal networks may be affected by altered gliotransmission initiated and maintained by tissue hypoxia and by the actions of inflammatory cytokines released by activated microglia.

There is morphological evidence of glial abnormalities in the brains of hypertensive subjects (Tomassoni *et al*. 2004). In an attempt to ‘inhibit’ astrocytic activity in SHRs, non‐specific pharmacological agents, such as arundic acid, have been used. Arundic acid suppresses synthesis of S100B (Asano *et al*. 2005), a glia‐specific Ca^2+^‐binding protein important for the expression of pro‐inflammatory cytokines and control of apoptosis (Marenholz *et al*. 2004). Interestingly, treatment of SHRs with arundic acid resulted in a significant reduction in the arterial blood pressure and prevented hypertension‐related strokes (Higashino *et al*. 2009).

Previous studies have also revealed an association of events that may link microvascular inflammation and brain hypoxia with astroglial activation of presympathetic networks. Experiments using RT‐PCR showed increased expression of a pro‐inflammatory molecule, junctional adhesion molecule‐A (JAM‐A), in the endothelial cells of arterioles supplying the dorsal brainstem structures of both young (pre‐hypertensive) and adult SHRs (Waki *et al*. 2007). Overexpression of JAM‐A in the dorsal medullary regions using viral vectors led to a significant increase in systolic blood pressure in control (Wistar) rats, and this was accompanied by leucocyte adhesion to the brainstem microvasculature (Waki *et al*. 2007, 2008). Moreover, body‐wide downregulation of JAM‐A using a specially formulated ‘*vivo*’ morpholino delayed development of hypertension in the SHR (Xu *et al*. 2012). There is also evidence that infiltration of monocytes in the brain microvasculature triggers the synthesis of cytokines, including monocyte chemoattractant protein‐1, by astrocytes (Andjelkovic *et al*. 2000). Therefore, leucocyte adhesion to the brainstem microvasculature may result in astroglial activation and upregulation of cytokine expression (Waki *et al*. 2008). In addition, increased leucocyte adhesion may produce partial blood flow obstruction, resulting in increased vascular resistance, reduced tissue perfusion and hypoxia (Paton & Waki, 2009). This may be sufficient to trigger astroglial activation, inflammation and gliotransmitter (ATP) release, leading to increased excitability of presympathetic circuits and enhanced central sympathetic drive in neurogenic hypertension.

## Conclusion

The active role of glial cells in the central nervous mechanisms that maintain cardiovascular homeostasis in physiological and pathological conditions is a novel concept, and the intimate details of neuroglial interactions in the brainstem are only starting to be explored. The experimental evidence reviewed here identifies two potential mechanisms of how glial cells may impart their activity on presympathetic CNS networks (both hypothalamic and brainstem), leading to maladaptive and detrimental increases in sympathetic activity associated with the development and progression of heart failure and hypertension, as follows: (i) a decrease in cerebral O_2_ content that causes hypoxia‐induced astroglial activation (Fig. 1); and (ii) increased production of ATP (Fig. 1) and pro‐inflammatory cytokines by activated astrocytes and microglia. Further advances in our understanding of micro‐ and astroglial function will not only reveal the role of these cells in modulating the activities of vital presympathetic networks that control the heart and the vasculature, but may also help to identify novel therapeutic modalities for the treatment of cardiovascular disease.

## Additional information

### Competing interests

None declared.

### Author contributions

A.V.G. and N.M. drafted the manuscript. A.G.T. and S.K. revised the manuscript critically for intellectual content. All authors approved the final version of the manuscript and agree to be accountable for all aspects of the work in ensuring that questions related to the accuracy or integrity of any part of the work are appropriately investigated and resolved. All persons designated as authors qualify for authorship, and all those who qualify for authorship are listed.

### Funding

The research in our laboratories referred to in this report was funded by The Wellcome Trust and the British Heart Foundation. N.M. is a British Heart Foundation Intermediate Basic Research Science Fellow FS/13/5/29927. A.V.G. is a Wellcome Trust Senior Research Fellow. S.K. is funded by MRC MR/L020661/1 and BBSRC BB/L019396/1.
